# Serum TNF-α level and probing depth as a combined indicator for peri-implant disease

**DOI:** 10.1590/1414-431X2023e12989

**Published:** 2024-01-22

**Authors:** Huerxidai Yilihamujiang, Xiaofeng Ni, Mingkai Yu, Shuya Dong, Long Mei, Yuxiang Zheng, Lujin Cheng, Nannan Pang

**Affiliations:** 1 Department of Prosthodontics and Dental Implant, Xinjiang Medical University Affiliated First Hospital, School of Stomatology, Xinjiang Uyghur Autonomous Region, Urumqi, China; 2 Stomatological Research Institute of Xinjiang Uygur Autonomous Region, Urumqi, China; 3 School of Public Health, Xinjiang Medical University, Urumqi, China; 4 Department of Pathology, the First Affiliated Hospital of Shihezi University, Shihezi, Xinjiang, China

**Keywords:** Peri-implant disease, Cytokines, Biomarker, Clinical parameters

## Abstract

Peri-implant disease (PID) is a general term for inflammatory diseases of soft and hard tissues that occur around implants, including peri-implant mucositis and peri-implantitis. Cytokines are a class of small molecule proteins, which have various functions such as regulating innate immunity, adaptive immunity, and repairing damaged tissues. In order to explore the characteristics and clinical significance of tumor necrosis factor (TNF)-α, interleukin (IL)-6, IL-10, and tumor growth factor (TGF)-β1 expression levels in serum of patients with peri-implant disease, 31 patients with PID and 31 patients without PID were enrolled. The modified plaque index (mPLI), modified sulcus bleeding index (mSBI), and peri-implant probing depth (PD) were recorded. The levels of serum TNF-α, IL-6, IL-10, and TGF-β1 were detected by ELISA. TNF-α, mPLI, mSBI, and PD levels were significantly higher in the PID group. TGF-β1 levels were significantly higher in the control group. There was a significant positive correlation between TNF-α and mPLI, mSBI, and PD. TGF-β1 was negatively associated with TNF-α, mPLI, mSBI, and PD. Multiple logistic regression analysis showed that TNF-α and PD were risk factors for the severity of PID. The receiver operating curve analysis showed that high TNF-α levels (cut-off value of 140 pg/mL) and greater PD values (cut-off value of 4 mm) were good predictors of PID severity with an area under the curve of 0.922. These results indicated that TNF-α and PD can be used as a biological indicator for diagnosing the occurrence and progression of PID.

## Introduction

With the rapid development of oral implantology and the increasing use of implants, the incidence of peri-implant diseases (PID) is showing an increasing trend (1-6). PID is one of the common complications of oral implantation and an important reason for the failure of implant treatment. Therefore, it is very important to find the lesions around the implant and perform intervention early. In recent years, stomatologists have been committed to the study of the pathogenesis of periodontal disease. In their immune studies, it was found that tumor necrosis factor (TNF)-α, interleukin (IL)-6, IL-10, and tumor growth factor (TGF)-β1 were closely related to the occurrence and development of periodontitis (7-9). PID has shown great similarity with periodontitis in many aspects, but also has some differences (10). Although some studies have confirmed the existence of various cytokines around the implant, there are few immunological studies on the effect of cytokines on PID (11,12). To further explore the occurrence and development of PID, this study explored the cytokines that are closely related to periodontal disease. It has been documented that inflammatory cytokines can be used as indicators for early diagnosis of PID (13). Bielemann et al. (14) tested cytokines in PID gingival crevicular fluid and concluded that IL-10 may be an immunological index before and after implantation. However, most of the cytokines in these studies came from gingival crevicular fluid around the implant. Although the gingival crevicular fluid is easy to obtain repeatedly, the low concentration of cytokines in the fluid may affect the experimental results. Therefore, we explored the levels of TNF-α, IL-6, IL-10, and TGF-β1 in peripheral blood combined with their clinical indicators of PID. The following questions were raised: During the process of PID occurrence and development, how are these cytokines expressed in peripheral blood and what kind of role do they play? Will they inhibit or promote the disease? Which cytokines are most closely associated with the disease process and may influence the outcome of the disease? This study aimed to provide immunological research basis and theoretical basis for the analysis of the pathogenesis of PID while providing new information for the early prevention and intervention of PID in clinical practice.

## Material and Methods

### Participants and setting

A total of 623 patients admitted to the Department of Xinjiang Medical University from January 2021 to March 2023 were selected for the prospective study. All patients were asked to attend the follow-up at 3, 6, and 12 months after implantation to observe the soft and hard tissues around the implant. Diagnostic and inclusion criteria (15-18) were no hypertension, heart disease or diabetes; no allergy or other autoimmune diseases; no smoking or drinking habits; not pregnant or lactating; and no pharmacological treatments such as non-steroidal drugs and immunosuppressants in the 6 months prior to inclusion. Patients in the PID group also had to meet the following criteria: the implant was functional for more than one year; the defect was located in the posterior dental region; only one implant in the mouth; and the lost tooth was a premolar or molar. A total of 31 patients with PID were selected. The control group consisted of 31 patients without PID for 12 months selected randomly. Peripheral blood was collected in both the PID group and the control group within 6 to 12 months after implantation. All patients gave informed consent.

### Clinical parameters

The clinical parameters assessed were: 1) modified plaque index (mPLI): 0=no plaque; 1=plaque can be found on the surface of the prosthesis; 2=visible plaque; 3=large amount of soft scale; 2) modified sulcus bleeding index (mSBI): 0=no bleeding; 1=punctate hemorrhage; 2=linear bleeding inside gingival crevicular area; 3=spontaneous bleeding; 3) probing depth of peri-implant pocket (PD) in mm. A special blunt plastic probe was used to avoid scratches on the surface of the titanium implant. A pressure of <0.25 N was used during probing so as not to destroy the tissue surrounding the implant (18). The probe was inserted into the peri-implant pocket along the long axis of the tooth at the mesial, median, and distal parts of the buccal or lingual side of the implant. The depth from the gingival margin to the pocket bottom was measured, and the average value of the six sites was considered. Probing examination included the presence of bleeding on probing (BOP), discharge of pus, and probing depth. Bleeding is a sign of inflammation of the soft tissue surrounding the implant. Exploratory pyorrhea is sometimes present, which is also characteristic of peri-implant inflammation.

### Diagnostic criteria

In accordance with the expert consensus of Mombelli et al. in 1995 (19), patients with the following diagnostic criteria were included: 1) healthy peri-implant: no inflammation in the peri-implant tissue, no bleeding after probing, and PD did not increase over time (19); 2) peri-implant mucositis: probing bleeding and/or pus discharge, 2≤ PD ≤5 (20) and; 3) peri-implantitis: bleeding with gentle probing and/or pyorrhea, PD ≥6 mm (19).

### ELISA

Serum samples were collected. After centrifugation for 10 min (1100 *g*/min, 4°C) the supernatant was collected and stored at -80°C. The levels of TNF-α, IL-6, IL-10, and TGF-β1 were detected by an ELISA kit (Jianglaibio, China), and the experimental procedures were carried out in accordance with the manufacturer's instructions. The absorbance of each well was measured with a microplate reader (Therma Fisher, USA) at a wavelength of 450 nm.

### Statistical analysis

All data were statistically analyzed using SPSS 25.0 (IBM, USA). The measurement data of normal distribution are reported as means±SD. The *t*-test was used for the comparison of independent samples, one-way analysis of variance was used for the comparison between multiple groups, and multivariate logistic regression was used to analyze the relevant factors. P<0.05 was considered as a statistically significant difference.

## Results

### Basic information, cytokine levels, and clinical parameters

**Figure 1:**
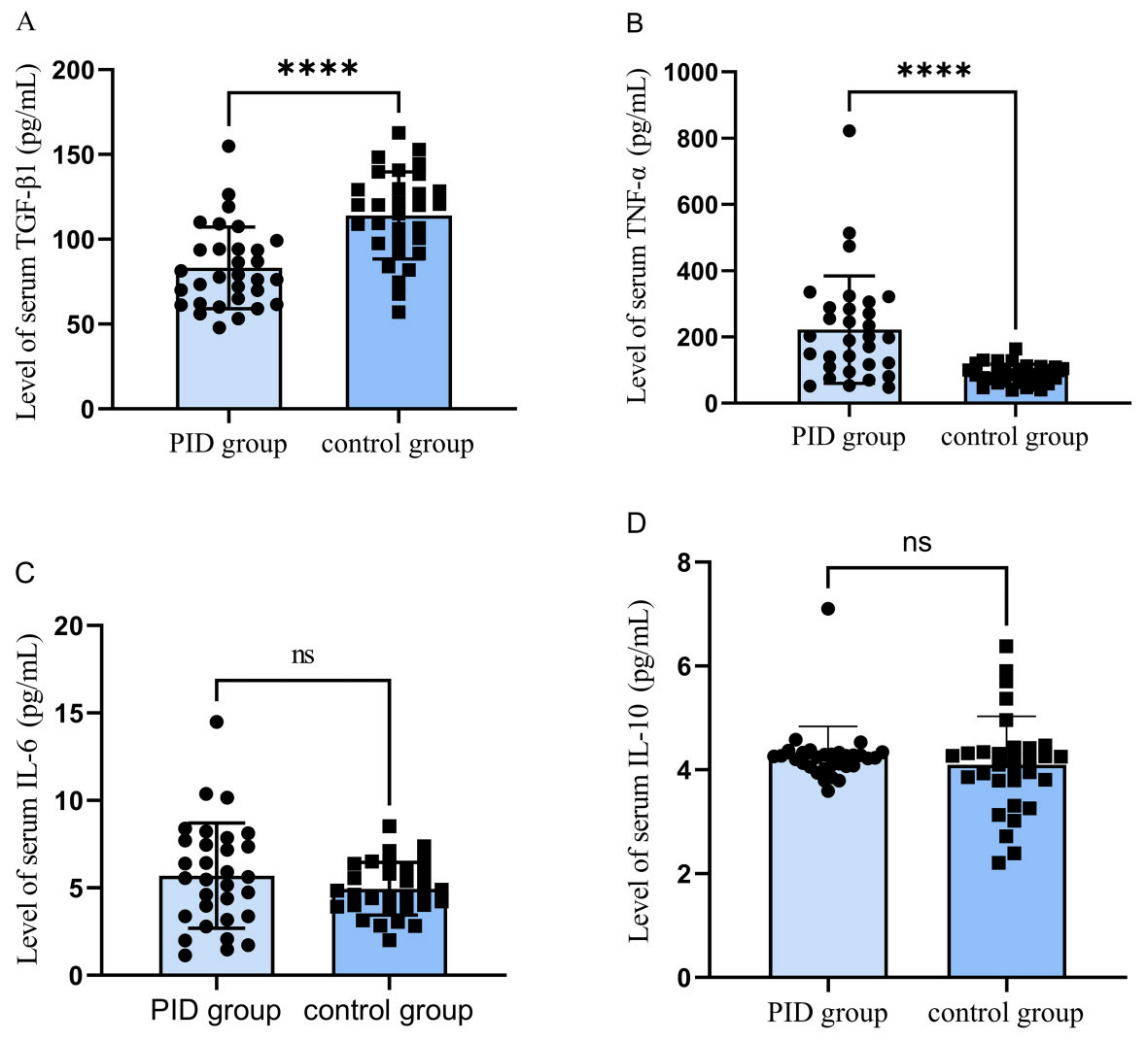
Serum levels of tumor growth factor (TGF)-β (**A**), tumor necrosis factor (TNF)-α (**B**), interleukin (IL)-6 (**C**), and IL-10 (**D**) in the peri-implant disease (PID) and control groups tested by ELISA. ****P<0.0001, Student's *t*-test. ns: No significant difference.

The 31 patients in the PID group were aged between 35 and 59 years, with men accounting for 70% (22 patients), and the 31 patients in the control group were aged between 36 and 52 years, and 55% (17 patients) were male (Table 1). The expression level of TNF-α in the peripheral blood was significantly higher in the PID group than in the control group (P<0.001), and on the contrary, the expression of TGF-β1 in the PID group was significantly lower than in the control group (P<0.001). The expression level of IL-6 was slightly higher in the PID group than in the control group, and IL-10 levels were essentially the same in the PID group and control group, with no statistical difference (P>0.05). In the PID group, the three clinical parameters, mPLI, mSBI, and PD, were higher than those in the control group (P<0.001) (Figure 1).

**Table 1:** Comparison of age, sex, mPLI, mSBI, and PD in the peri-implant disease (PID) and control groups.

	PID group	Control group	*t*/x^2^	P
Age (years)	47.61±12.89	44.06±8.24	1.29 (*t*)	0.202
Gender (M/F)	22/9	17/14	1.73 (x2)	0.189
mPLI	2.00±0.83	2.00±0.81	3.86 (*t*)	<0.001
mSBI	2.00±0.89	1.00±0.80	4.78 (*t*)	<0.001
PD	5.00±1.83	3.00±0.70	5.68 (*t*)	<0.001

Data are reported as mean and SD, except for gender. T-test (*t*) or chi-squared (x^2^) test was used. mPLI: modified plaque index; mSBI: modified sulcus bleeding index; PD: probing depth.

### Correlation analysis between cytokines and clinical parameters

The above results suggested that cytokines in the serum of PID patients were altered, and we further found that TNF-α levels were significantly positively correlated with mPLI (r=0.556), mSBI (r=0.588), PD (r=0.748) (P<0.001), and with IL-6 (r=0.336, P<0.001). TGF-β1 levels were negatively correlated with TNF-α, mPLI (r=0.488), mSBI (r=0.474), and PD (r=0.399) (P<0.001). A significant positive correlation was found between mPLI and mSBI (r=0.921, P<0.001), between mSBI and PD (r=0.557, P<0.001), and between mPLI and PD (r=0.491, P<0.001) (Table 2).

**Table 2:** Correlation between cytokine expression levels and clinical parameters.

	TNF-α	IL-6	IL-10	TGF-β1	mPLI	mSBI	PD
TNF-α	1	0.336**	-0.137	-0.439**	0.556**	0.588**	0.748**
IL-6	0.336**	1	-0.124	-0.242	0.362**	0.364**	0.457**
IL-10	-0.137	-0.124	1	0.107	-0.077	-0.044	-0.160
	-0.439**	-0.242	0.107	1	-0.488**	-0.474**	-0.399**
mPLI	0.556**	0.362**	-0.077	-0.488**	1	0.921**	0.491**
mSBI	0.588**	0.364**	-0.044	-0.474**	0.921**	1	0.557**
PD	0.748**	0.457**	-0.160	-0.399**	0.491**	0.557**	1

*P<0.05, **P<0.001 (bivariate correlation test). TNF-α: tumor necrosis factor α; IL: interleukin; TGF-β1: tumor growth factor β1; mPLI: modified plaque index; mSBI: modified sulcus bleeding index; PD: probing depth.

### Cytokine expression levels and clinical parameters in relation to PID

Logistic stepwise regression analysis was performed to assess the association between age, sex, the four cytokines, and clinical markers and PID. In univariate analysis, TNF-α, mPLI, mPLI, and PD were risk factors for PID (P<0.001), and TGF-β1 may be a protective factor for PID. Multiple logistic regression analysis showed that TNF-α and PD were two independent risk factors for PID (P<0.05) (Table 3).

**Table 3:** Univariate and multivariate logistic stepwise regression analyses of age, gender, cytokine levels, and clinical parameters.

	OR (95%Cl)	Univariate			OR (95%CI)	Multivariate	
		Wald	P-value			Wald	P-value
Age	1.032 (0.983-1.082)	1.634	0.201				
Gender	0.497 (0.174-1.419)	1.707	0.191				
TGF-β1	0.954 (0.930-0.978)	13.764	**<0.001**		0.972 (0.940-1.006)	2.640	0.104
TNF-α	1.029 (1.012-1.046)	10.968	**<0.001**		1.029 (1.003-1.055)	4.798	**0.028**
IL-6	1.149 (0.919-1.437)	1.49	0.222				
IL-10	1.343 (0.681-2.649)	0.724	0.395				
mPLI	3.144 (1.583-6.243)	10.712	**0.001**		0.385 (0.031-4.741)	0.554	0.457
mSBI	3.728 (1.866-7.448)	13.887	**<0.001**		2.159 (0.193-24.176)	0.390	0.532
PD	5.842 (2.158-15.816)	12.067	**<0.001**		6.873 (1.559-30.305)	6.483	**0.011**

Bold type indicates statistically significant. TGF-β1: tumor growth factor β1; TNF-α: tumor necrosis factor α; IL: interleukin; mPLI: modified plaque index; mSBI: modified sulcus bleeding index; PD: probing depth.

### Predictive analysis

**Figure 2:**
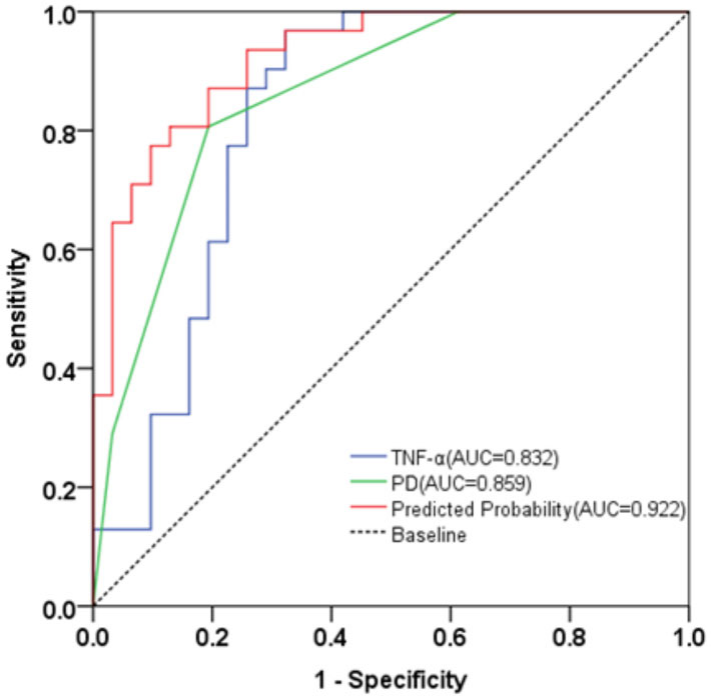
Receiver operating characteristic curve analysis for diagnostic value of tumor necrosis factor (TNF)-α and probing depth (PD) for peri-implant disease.

Receiver operating characteristic (ROC) curves were used to observe the predictive value of TNF-α and PD in the diagnosis of PID. TNF-α had an area under the curve (AUC) of 0.825; PD had an AUC of 0.859; and predicted probability had an AUC value of 0.922 (Figure 2). The cut-off values of TNF-α and PD were 140.07 pg/mL and 4 mm (Table 4). Spearman correlation analysis with TNF-α levels divided into ≥140.070 and <140.070 pg/mL showed that higher TNF-α levels were positively correlated with greater severity of the clinical parameters mSBI (P<0.05, r=0.644) and PD (P<0.05, r=0.513); there was no correlation with the clinical parameter mPLI (P>0.05). The above results indicated that the higher the serum TNF-α level in PID patients, the more serious the alveolar bone destruction and the worse the prognosis (Table 5).

**Table 4:** Area under the curve (AUC) analysis for tumor necrosis factor (TNF)-α and probing depth (PD) to predict the activity of peri-implant disease.

	AUC (95%CI)	P	Cutoff	Sensitivity	Specificity
TNF-α	0.832 (0.722-0.943)	0.000	140.070	0.677	1.0
PD	0.859 (0.766-0.952)	0.000	4.000	0.806	0.806
PP	0.922 (0.858-0.986)	0.000	0.558	0.742	0.935

PP: Predicted probability.

**Table 5:** Subgroup analysis for the effect of tumor necrosis factor (TNF)-α on risk of peri-implant disease activity.

	TNF-α <140.07 (n)	TNF-α ≥140.07 (n)	x^2^	P	r
mPLI					
**<**2	17	5	1.891	0.169	0.588
≥2	24	16			
mSBI					
**<**2	23	6	4.226	**0.04**	**0.644**
≥2	18	15			
PD					
**<**4	26	5	8.713	**0.003**	**0.513**
≥4	15	16			

Bold type indicates statistically significant (chi-squared test). mPLI: modified plaque index; mSBI: modified sulcus bleeding index; PD: probing depth.

## Discussion

PID includes peri-implant mucositis (PIM) and peri-implantitis (PI). PIM refers to the inflammatory lesions of the peri-implant mucosa, and PI refers to the peri-implant mucosal inflammation associated with loss of supporting bone (18). Several studies have shown that IL-6, IL-10, TNF-α, and TGF-β1 are closely related to the occurrence and development of PID (21). IL-6 is a pro-inflammatory factor that promotes the growth and differentiation of osteoclast precursors and alveolar bone resorption (22), and IL-10 is an important regulator of the pro-inflammatory process, downregulating the synthesis of Th1 pro-inflammatory cytokines and preventing the occurrence of excessive inflammation by acting on macrophages (23). TNF-α can be produced and released by a variety of cells in the body, with monocyte/macrophages being its main source (23), and it is one of the key cytokines regulating alveolar bone reconstruction (24). TGF-β1 is considered an important regulator of bone growth (24,25), promoting osteoblast proliferation and differentiation and fibroblast secretion of fibrin and collagen, and inhibiting osteoclast activity (26).

Several studies have shown that IL-1β, IL-6, IL-17, and TNF-α are the most common pro-inflammatory mediators associated with PID (27). In this study, the expression level of TNF-α in the PID group was significantly higher than the control group, and there was a strong correlation with mPLI, mSBI, and PD; higher TNF-α above 140 ng/L combined with the clinical parameter PD could predict the occurrence of PID. High expression of TNF-α may promote the progression of PID to a certain extent. In this study, there was no significant difference in IL-6 and IL-10 levels in peripheral blood of PID and non-PID patients, possibly because a rapid increase of IL-6 correlates with inflammation caused by extensive trauma (28), but the initiating factor for PID was oral plaque. The role of anti-inflammatory factor IL-10 as an inhibitor in the occurrence and development of PID has not been discussed in detail and needs further study. Vehof et al. (29) tested the level of TGF-β1 in the blood of implant patients and found that TGF-β1 increased significantly within 4 months of implantation, and then gradually decreased. In this study, serum TGF-β1 level was detected 6 months after implantation and restoration, and the level in the PID group was significantly lower than that in the control group, i.e., it failed to exert immunosuppression.

Due to the invasion of pathogens and the imbalance of the host's autoimmune response, inflammatory diseases occur in the tissues around the implant, with an increase of pro-inflammatory factors and an decrease of anti-inflammatory factors, eventually leading to the occurrence of PI or only PIM or no clinical manifestations (30-32). In order to observe the relationship between inflammation-related cytokines and clinical parameters, the correlation analysis was further carried out, and the results showed that IL-6, TNF-α, and TGF-β1 had a strong correlation with clinical parameters in the peripheral blood of PID patients. The expression levels of TNF-α and IL-6 in peripheral blood were directly proportional, each with different effects on alveolar bone resorption. The expression levels of TNF-α and TGF-β1 were negatively correlated, indicating that if TNF-α was released continuously, the expression of inflammation will increase; if the release of TNF-α was reduced, the increase of TGF-β1 will alleviate the inflammatory response. In addition, this study also found a strong positive correlation between mPLI, mSBI, and PD, indicating that when PID occurs, these three parameters will increase, and the higher the index, the more serious the degree of inflammation. At this time, the level of pro-inflammatory cytokines in the body will also increase with the development of inflammation.

Single factor and multifactor logistic regression analysis found that TNF-α and PD were independent risk factors for PID. TGF-β1 and mSBI were statistically significant in the univariate analysis, but not in the multivariate analysis. It is noteworthy that PD is the only clinical parameter in the multivariate analysis that can reflect the resorption of alveolar bone around the implant. In the follow-up visit after implantation, clinical parameters are the most commonly used criteria by doctors. If a patient has increased mPLI and light BOP around the implant, with or without an increase in PD from the baseline level, the case is diagnosed as PIM (33), which is reversible. If there is no resorption of alveolar bone, no specific treatment is needed other than instructing the patient to pay attention to oral hygiene. However, if it is allowed to develop, it will become irreversible, thus leading to PI.

Based on the results of subgroup analysis of TNF-α levels and data from relevant literature, low concentrations of TNF-α in the early stage after transplantation can indirectly promote the expression of osteoblast proteins such as osteocalcin, promote the differentiation and maturation of osteoblasts, and cause new bone formation. High concentrations of TNF-α promote the maturation and differentiation of osteoclasts and cause bone resorption (34,35). This shows the dual effects of TNF-α in osteogenesis and osteoclast activity (36). This study speculates that the best time for starting treatment is when a patient's PD is about 4 mm and its TNF-α expression level is high. PD could be used as a clinical parameter to determine whether TNF-α is highly expressed.

Finally, through ROC analysis, it was found that TNF-α and PD were helpful for the prediction of PID, and when TNF-α and PD were detected together, the accuracy of predicting PID was higher than either one alone.

This study provides new information for diagnosis, treatment, and prevention of PID based on cytokines, and could help dentists determine whether peri-implant maintenance is required during follow-up visits. However, this study evaluated only four of the cytokines most commonly associated with PID. Further investigation on whether PD can be a clinical indicator for increased TNF-α levels seems promising. Studies with a larger sample size should continue to collect peripheral blood from PID patients and measure changes in PD at different concentrations of TNF-α. Other inflammatory markers that can also play a role in the healing process and interact synergistically with the analyzed cytokines should be included in future studies.
